# Angioedema suppressed by a combination of anti-histamine and leukotriene modifier

**DOI:** 10.1186/s13223-017-0201-1

**Published:** 2017-06-13

**Authors:** Brendan N. Wong, Peter Vadas

**Affiliations:** Division of Allergy and Clinical Immunology, Department of Medicine, St. Michael’s Hospital, Toronto, Canada

**Keywords:** Angioedema, Urticaria, Histaminergic, Non-histaminergic, Anti-histamines, Leukotriene receptor antagonist, Cetirizine, Montelukast

## Abstract

**Rationale:**

Angioedema without co-existent urticaria is due to a limited number of causes, including hereditary and acquired C1 esterase inhibitor deficiency, drug-induced angioedema or idiopathic histaminergic or non-histaminergic angioedema. We describe a cohort of patients with recurrent angioedema whose clinical features and response to medications are distinct from the causes above.

**Methods:**

Patients were accrued retrospectively from an academic allergy practice between 2007 and 2014. After institutional research ethics board approval, patients’ charts were reviewed and demographic, clinical and laboratory data were extracted.

**Results:**

A total of 11 patients were recruited. The mean age at presentation was 54.9 years (range 19–70 years) and 6 of 11 were male. The mean number of episodes per year was 18.7 (range 2–60) and mean duration of episodes was 22.4 h (range 4–96). About half of episodes (52%) began overnight. Areas of involvement were lips (73%), tongue (64%), eyelids (18%), feet (36%) and hands (27%). None of the patients had low C3, C4, or CH50; none had significantly positive ANA; C1 esterase inhibitor level and function and C1q were normal in all patients tested. In these 11 patients, complete suppression of recurrences by the combination of cetirizine 20 mg daily and montelukast 10 mg daily was reported by 9 (82%) of patients; whereas 2 (18%) of patients had a partial response to this combination of medications.

**Conclusions:**

Herein, we report a form of angioedema without urticaria, mediated by a combination of histamine and leukotrienes. Clinical, demographic and therapeutic characteristics differentiate this from other recognized causes of angioedema.

## Background

Angioedema is the non-pitting swelling of the subcutaneous or submucosal tissues, caused by a transient increase in capillary permeability. Episodes of swelling usually last from a few hours to several days, and frequency of recurrences may vary from a few times per week to a few times per year.

Angioedema has been classified into two broad categories: with or without co-existent urticaria. Acute episodic angioedema with urticaria is relatively commonplace. Recognized causes include chronic spontaneous and inducible urticaria and reactions to foods, venoms, latex, drugs, or physical triggers [1]. In contrast, angioedema without urticaria is considerably less common, occurring 2.4% of the time [2]. Angioedema without urticaria may be due to congenital C1 esterase inhibitor deficiency [type I hereditary angioedema (HAE)], dysfunctional C1 esterase inhibitor (type II HAE) and hereditary angioedema with normal C1 inhibitor and mutations in the factor XII gene [1, 3, 4]. Kinin-mediated angioedema associated with angiotensin converting enzyme inhibitors and direct renin inhibitors presents without urticaria [1]. HAE, acquired C1 esterase inhibitor deficiency and drug-induced angioedema, have been well characterized and the mechanisms and mediators are elucidated.

After having ruled out the causes above, more than one-third of cases of angioedema are classified as idiopathic. Histaminergic and non-histaminergic angioedema are two distinct forms of idiopathic angioedema that have been characterized thus far in the literature. In a large cohort of 776 patients with angioedema but without urticaria, 16% were caused by drugs, insect stings or foods; 11% due to angiotensin converting enzyme inhibitors; 7% due to autoimmune disease or infection and 25% due to C1 esterase inhibitor deficiency [5]. In the remaining 41% of cases, 33% had idiopathic histaminergic and 5% had idiopathic non-histaminergic angioedema. Whereas histaminergic angioedema is suppressed by first and second generation H1 anti-histamines, idiopathic non-histaminergic angioedema may be responsive to other agents, such as steroids, dapsone, rituximab and icatibant [6–8].

Herein, we describe a form of recurrent angioedema which appears to be distinct from histaminergic and non-histaminergic forms in that it is responsive to a combination of anti-histamine and leukotriene receptor antagonist (LRA).

## Methods

A cohort of 11 patients was accrued retrospectively from an academic allergy practice from 2007 to 2014. Following institutional research ethics board approval, patients’ charts were reviewed and demographic, clinical, and laboratory data were extracted. Patients were included in this study when other recognized causes of angioedema were ruled out by history, laboratory investigations, and whose angioedema was either completely or partially suppressed by a combination of antihistamine and leukotriene receptor antagonist.

The location, timing, frequency and clinical characteristics of angioedema were elicited. Family history of angioedema, medication use, relevant exposures prior to recurrences, potential triggers such as infection, stress, trauma and others were recorded. Laboratory investigations included C3, C4, CH50 and C1q complement studies, anti-nuclear antibody (ANA), C1 esterase inhibitor antigen level, and C1 esterase inhibitor functional assay determinations.

## Results

A total of 11 patients with angioedema were identified whose characteristics fulfilled the criteria above. There were 6 males (55%) and 5 females (45%). Mean age of onset of angioedema occurred at 51.7 years (range 13–64 years). Mean age at first assessment was 54.9 years (range 19–70 years).

Mean number of episodes per year was 18.7 (range 2–60 episodes), and 52.0% (range 0–100%) of those episodes began overnight. Mean duration of those episodes was 22.4 h (range 4–96 h).

Anatomic localization of angioedema was as follows: 73% of patients had swelling of the lips, 64% of the tongue, 45% of the cheeks, 36% of the feet, 27% of the hands, 18% of the eyelids, 18% of genitalia, 18% of the neck, 18% of the throat and 18% of the face.

In 4 of 11 patients, the angioedema was bland (36%), whereas 7 of 11 (64%) experienced pareasthesia.

Whereas 2 patients were taking angiotensin-converting-enzyme (ACE) inhibitors (18%), 1 patient was taking an angiotensin II receptor blocker (9%) and 1 patient was on a direct renin inhibitor (9%), discontinuation of these medications did not lead to resolution of recurrent angioedema after a minimum of 6 months. None of the 11 patients in this study had a family history of angioedema.

Laboratory investigations were performed in all patients to rule out connective tissue diseases and C1 esterase inhibitor deficiency. 11 patients (100%) had negative antinuclear antibody (ANA) screening assays. All patients (100%) had normal levels of C3, C4, CH50, C1q, C1 esterase inhibitor levels and C1 esterase inhibitor functional assays.

All 11 patients consented to suppressive therapy, These 11 patients were initially treated with high dose cetirizine (40 mg daily). None of the 11 patients treated with cetirizine alone reported complete suppression of angioedema. These 11 patients were then treated with a combination of cetirizine 20 mg once daily and montelukast 10 mg once daily. Of these 11 patients, 9 patients (82%) had complete suppression using a combination of cetirizine 20 mg daily and montelukast 10 mg daily; and the remaining 2 of 11 patients (18%) given cetirizine and montelukast had partial suppression (50 and 66% respectively) of recurrences.

## Discussion

Idiopathic angioedema is generally classified into either histaminergic or non-histaminergic forms. The idiopathic histaminergic form responds to treatment with antihistamine monotherapy. Idiopathic non-histaminergic angioedema may respond to a variety of medications, including steroids, dapsone, rituximab, icatibant (a bradykinin receptor antagonist), ecallantide (a kallikrein inhibitor), C1 esterase inhibitor concentrate, tranexamic acid, cannabis and omalizumab [6–13]. Neither group has distinguishable laboratory features [2]; however, the two groups differ in demographics and clinical features.

Idiopathic histaminergic angioedema has no sex predilection and tends to occur equally in males and females. Attacks of idiopathic histaminergic angioedema respond to antihistamines for both acute treatment and long-term suppression [5]. The mean age of presentation is 50 years, with mean duration of attacks of 28.1 h [14]. Upper airway involvement was reported in 54.8% and tongue swelling in 29% of patients. All patients responded to antihistamine therapy.

Idiopathic non-histaminergic angioedema tends to occur more often in males, with the age of onset beginning at a considerably younger age. Episodes may also affect cutaneous sites and abdominal organs, but are not known to affect the lips and tongue. Mansi et al. described a large cohort of 1058 patients with angioedema without urticaria [15]. Of these, 377 had hereditary and 681 acquired angioedema. 49 of 681 had acquired C1 esterase inhibitor deficiency and 183 had angioedema secondary to angiotensin converting enzyme inhibitors. Idiopathic histaminergic angioedema was diagnosed in 379 patients and idiopathic non-histaminergic angioedema was diagnosed in 70 patients. The median age of onset of non-histaminergic angioedema was 38 years, with a median duration of attacks of 48 h. More than 80% of patients with non-histaminergic angioedema reported more than 5 attacks per year. Attacks were reported in the face in 86%; tongue and oropharynx (55%); extremities (56%) and gastrointestinal tract in 20%. A response to tranexamic acid was seen in 37 of 38 patients who consented to treatment [15].

In the cohort described in our series, the angioedema seems to be mediated by a combination of histamine and leukotrienes, in that partial or complete suppression of recurrences is seen by using a combination of receptor antagonists to these two mediators. The prevalence of this form was equal in males and females, with a mean age of onset of 52 years. The mean duration of attacks was 22.4 h. Most patients had involvement of the head and neck, 18% had involvement of the upper airway. None of our patients had abdominal attacks. Angioedema was not suppressed by high dose antihistamine monotherapy, whereas the combination of antihistamine and montelukast was effective. This form of angioedema often begins unprovoked overnight, possibly due to the nocturnal drop in endogenous cortisol.

The clinical course of angioedema is not benign, in that significant numbers of patients may experience upper airway involvement, although none of the patients required intubation for airway compromise. Nevertheless, patients diagnosed with this form of angioedema should be provided with an epinephrine auto-injector.

Angioedema responsive to a combination of anti-histamine and leukotriene modifier drugs does not imply a previously unreported form of angioedema, but does suggest that a combination of histamine and leukotrienes may contribute to recurrences of angioedema in some patients. Whether angioedema in these patients is unique and distinct from previously reported forms remains to be determined and will require further investigation. Specifically, non-histaminergic forms of angioedema responsive to other drugs, such as steroids, rituximab, tranexamic acid and dapsone will have to be investigated for their response to the combination of histamine and leukotriene antagonists.

## Conclusions

Angioedema without urticaria may be due to many causes, and a systematic approach to investigation and treatment is required. Where clinical evaluation and laboratory investigations have ruled out medications, autoimmunity, infection and C1 esterase inhibitor deficiency, idiopathic forms of angioedema may be considered. This latter category appears to have 3 subtypes: namely, idiopathic histaminergic angioedema, idiopathic non-histaminergic angioedema and idiopathic histamine- and leukotriene-mediated angioedema. This latter form of angioedema appears to be distinct from the other idiopathic types based on the clinical profile of affected patients and their response to suppressive therapy.
